# LncRNA NALT1 promotes colorectal cancer progression via targeting PEG10 by sponging microRNA-574-5p

**DOI:** 10.1038/s41419-022-05404-5

**Published:** 2022-11-16

**Authors:** Mengling Ye, Lu Zhao, Lu Zhang, Siyi Wu, Zhao Li, Yi Qin, Fei Lin, Linghui Pan

**Affiliations:** 1 Department of Experimental Research, Guangxi Medical University Cancer Hospital, Nanning, China; 2 Department of Anesthesiology, Guangxi Medical University Cancer Hospital, Nanning, China; 3 Guangxi Clinical Research Center for Anesthesiology, Nanning, China; 4 Guangxi Engineering Research Center for Tissue & Organ Injury and Repair Medicine, Nanning, China; 5 Guangxi Key Laboratory for Basic Science and Prevention of Perioperative Organ Disfunction, Nanning, China

**Keywords:** Colon cancer, Colon cancer

## Abstract

Colorectal cancer (CRC) is currently one of the commonest tumors and the main reason for cancer-related deaths worldwide. It has been reported that long non-coding RNAs (lncRNAs) act as important indicators and regulators in various cancers. There is an urgent need to explore new lncRNA biomarkers in CRC, as well as their functions and molecular mechanisms. NALT1 has been implicated in the occurrence of gastric cancer (GC). However, the detailed function and mechanism of NALT1 in CRC progress have not been reported. In this study, RT-qPCR was conducted to detect the expression of NALT1 in 76 CRC patients ranging from stages I through IV. To assess the biological function of NALT1, loss- and gain-of-function experiments were conducted both in vivo and in vitro. Moreover, RNA-seq, bioinformatics analysis, RNA pulldown assay, dual-luciferase reporter, Ago2-RIP, quantitative PCR, Western blot assays, and rescue experiments were performed to reveal the molecular mechanisms of competitive endogenous RNAs (ceRNAs). It was observed that high expression of NALT1 was markedly correlated with advanced cancer stage in the clinic. Functionally, NALT1 downregulation inhibited cell proliferation, migration and invasion, whereas NALT1 overexpression exhibited an opposite trend both in vivo and in vitro. Bioinformatics analysis, RNA pulldown, Ago2-RIP, and luciferase reporter assays showed that miRNA-574-5p was a target of NALT1. Additionally, dual-luciferase reporter assays, Ago2-RIP, and rescue experiments indicated that miRNA-574-5p could target the PEG10 gene directly. Our results suggested that NALT1 promoted CRC proliferation and migration by sponging miRNA-574-5p to upregulate PEG10 expression, and implied that NALT1 might act as a promising biomarker and therapeutic target for CRC.

## Background

Colorectal cancer (CRC) is the 3rd most common cancer and the 2nd leading cause of cancer-related mortality worldwide [1]. As the clinical symptoms of CRC are nonspecific in the early stages, so it is easy to be misdiagnosed and most of the cases are diagnosed at advanced disease stages. There is an increase in the frequency of recurrent metastatic CRC patients with advanced stage [2]. Hence, molecular mechanisms underlying CRC tumorigenesis and progression are of the utmost imperative to be explored.

Long noncoding RNA (lncRNA) is a transcript with >200 nucleotides that lack a protein-coding capacity [3]. It has received increasing attention because of its great biological significance in the occurrence and progression of cancers. LncRNA NOTCH1 associated lncRNA in T cell acute lymphoblastic leukemia 1 (NALT1) acts as a regulator to be implicated in the development of gastric cancer (GC) [4]. Additionally, dysregulated NALT1 expression was found to be associated with overall survival (OS) in stomach adenocarcinoma (STAD) and might facilitate cancer cell proliferation [5]. Dysregulation of lncRNAs has been commonly found in CRC, which may be involved in CRC cell growth, migration, and metastasis [6–8]. However, the relationship between NALT1 and CRC is yet to be explored.

The competing endogenous RNA (ceRNA) network is a post-transcriptional regulatory mechanism of lncRNAs, in which lncRNAs competitively bind miRNAs to regulate mRNAs [9]. Several lncRNAs have been shown to regulate the pathogenesis of CRC as tumor-suppressor genes or oncogenes via acting as ceRNAs [10, 11]. For example, lncRNA MIR17HG could act as a ceRNA to modulate HK1 expression by sponging miRNA-138-5p and promote CRC cell invasion and liver metastasis [11]. Therefore, it is necessary to further explore the mechanisms of NALT1 binding miRNAs in the ceRNA network in CRC.

Paternally expressed gene 10 (PEG10), a retrotransposon-derived imprinted gene, was located in human 7q21 [12]. Previous reports have reported that the PEG10 gene exhibits a high degree of tissue-specificity and contributes to tumor cell proliferation and invasion of CRC [13, 14]. For example, PEG10 promotes proliferation and suppresses apoptosis in CRC. Also, elevated PEG10 expression has been found in prostate cancer [15], endometrial cancer [16], and bladder cancer [17]. Besides, PEG10 participated in the lncRNA-mediated ceRNA regulated network in hepatocellular carcinoma [18]. In this article, RNA-sequencing revealed that PEG10 might be a target gene of NALT1. Thus, we assumed that PEG10 plays a vital role in the NALT1-mediated ceRNA network in CRC. To prove our hypothesis, we constructed the CRC cell lines and animal models and carried out relevant studies.

## Materials and methods

### Patients and tissue samples

A total of 76 patients who had undergone radical resection in Guangxi Medical University Affiliated Tumor Hospital from January 2016 to July 2019 were enrolled. All of these patients did not receive any chemotherapy or radiotherapy before resection. The tumor tissues were collected snap-frozen in liquid nitrogen immediately after removal and stored at −80 °C until use or paraffin embedded. This research has been approved by the Ethics Committee of Guangxi Medical University Affiliated Tumor Hospital and informed consent was obtained. Detailed information of these patients is presented in Table 1.

**Table 1:** Correlation between the expression levels of NALT1 and clinicopathological features in 76 CRC patients.

	*N* = 76	NALT1 expression		*P* value
clinicopathological parameters		Low	High	
		*n* = 38	*n* = 38	
Gender				
Male	38	20	18	0.6463552
Female	38	18	20	
Age				
<60	35	19	16	0.55519995
≥60	41	19	22	
Tumor location				
Colon	41	20	21	0.75990079
Rectum	35	18	17	
Differentiation				
Well and Moderate	70	36	34	**<0.001**
Poor	6	2	4	
Lymph node metastasis				
Absent	45	27	18	**0.0087101**
Present	31	11	20	
Distant metastasis				
Absent	57	30	27	**0.0085187**
Present	19	8	11	
Nerve Invasion				
Absent	45	23	22	0.34670665
Present	31	15	16	
Tumor size				
≤5 cm	56	30	26	**0.0068523**
>5 cm	20	8	12	
CEA level				
<5 μg/mL	44	22	22	0.49129712
≥5 μg/mL	32	16	16	

Bold values emphasize the difference between two or multiple groups which was statistically significant (*P* values < 0.05).

### Cell culture

Human CRC cells (HT29 and HCT116) were supplied by the Chinese Academy of Sciences (Shanghai, China); the normal human intestines epithelial cell line (NCM460) was obtained from Procell Life Science & Technology Co, Ltd. (Wuhan, China). HCT116 and HT29 cells were cultivated in DMEM medium (Invitrogen, USA) and NCM460 was cultivated in RPMI-1640 medium (Invitrogen, USA). The culture media were all supplemented with 10% FBS and 1% penicillin-streptomycin. The cells were maintained at 37 °C and 5% CO_2_.

### Real-time quantitative polymerase chain reaction

Total RNA was isolated with TRIzol reagent (Invitrogen, USA), and cDNA synthesis was then conducted. Real-time quantitative polymerase chain reaction (RT-qPCR) was conducted with SYBR Green Master Mixture (Vazyme, China). GAPDH was utilized as the internal control. The relative expression of each gene was measured using the 2^−ΔΔCt^ method. Each assay was repeated at least three times. The primer pairs employed for RT-qPCR are presented in Table 2.

**Table 2:** Sequences used in this study.

ID	Sequences
GAPDH forward	5′-CTCTGATTTGGTCGTATTGGGC-3′
GAPDH reverse	5′-CCTGGAAGATGGTGATGGGATT-3′
NALT1 forward	5′-TCATAAGCCCGCGTTACGG-3′
NALT1 reverse	5′-CCTTGAGCTGGCATCCTCATG-3′
PEG10 forward	5′-GGACCTGGATTGGAACGAG-3′
PEG10 reverse	5′-GAGCAGACAGCGACTTGG-3′
U6 forward	5′-CTCGCTTCGGCAGCACATATACT-3′
U6 reverse	5′-ACGCTTCACGAATTTGCGTGTC-3′
miR-574-5p forward	5′-CGCGTGAGTGTGTGTGTGTGA-3′
miR-574-5p reverse	5′-AGTGCAGGGTCCGAGGTATT-3′
NALT1 gRNA	5′-CACC GGACGTGGAGGCTGCGTAGG-3′
PEG10 siRNA1 forward	5′-CCCACUACCUGAUGCACAATT-3′
PEG10 siRNA1 reverse	5′-UUGUGCAUCAGGUAGUGGGTT-3′
PEG10 siRNA2 forward	5′-GCACUCGAUCUAUCGUCUUTT-3′
PEG10 siRNA2 reverse	5′-AAGACGAUAGAUCGAGUGCTT-3′
PEG10 siRNA3 forward	5′-GCCACUCUAUUAUCCAGUATT-3′
PEG10 siRNA3 reverse	5′-UACUGGAUAAUAGAGUGGCTT-3′

*gRNA* guide ribonucleic acid, *siRNA* small interfering RNA.

### sgRNA design and plasmid construction

The specific single-guide RNA (sgRNA) sequences targeting the third exon of the NALT1 gene (Gene ID: 101928483) were designed by an online tool (http://www.e-crisp.org/E-CRISP/designcrispr.html). The coupled complementary DNA oligos were annealed and inserted into the BsmBI sites of linearized LentiCRISPR v2 vector using T4 DNA ligase. The sequences of sgRNA NALT1 were 5′-CACCGGACGTGGAGGCTGCGTAGG-3′.

### Plasmids and transfection

The full length of NALT1was cloned with PCR and inserted into lentiviral vector pLEX-MCS. SiRNAs targeting PEG10 and miRNA-574-5p mimic/inhibitor were designed and synthesized by GenePharma (GenePharma, China) and RiboBio (Guangzhou, China), respectively. The full-length NALT1-WT and NALT1-mut (miRNA-574-5p) were amplified by PCR and subcloned into the psiCHECK-2 vector (Promega, USA). The 3′-untranslated region (3′-UTR) of PEG10 mRNA containing the intact miRNA-574-5p recognition sequences was amplified by PCR and subcloned into the psiCHECK-2 vector. The sequences of siRNA PEG1 were presented in Table 2.

HCT116 and HT29 cells were grown in 24-well or 6-well plates in a culture medium and transfected with plasmids, mimics, and inhibitors using Lipotransfectamine 3000 (Invitrogen, USA). Transfection of PEG10 siRNA or NC siRNA was conducted with Lipofectamine RNAiMAX (Invitrogen, USA). HCT116 and HT29 cells were infected with the lentiviral construct of NALT1 LentiCRISPR v2 or pLEX-MCS and their control vectors and cultured in DMEM medium with 4 mg/mL puromycin (Invitrogen, USA).

### Cell counting kit-8 assay

Cell viability was examined by Cell counting kit-8 (CCK-8) assay. HCT116 or HT29 cells (1 × 10^3^ cells/well) were grown in 96-well plates. After transfection for 24, 48, and 72 h, CCK-8 solution (Vazyme, China) was added to each well by following the kit’s protocol.

### Transwell assay

For migration assays, the transfected CRC cells were transferred into the upper chamber without Matrigel (Corning, NY, USA) and cultured in DMEM medium containing 0.1% FBS. For invasion assays, cells were seeded into the upper chambers coated with Matrigel (Corning, NY, USA). Meanwhile, 10% FBS-containing DMEM medium was placed in the bottom chamber. After 36 h, the chambers were fixed with 4% paraformaldehyde (Beyotime, China) and stained with 0.1% crystal violet (Solarbio, China). CarlZeiss inverted microscope was used to observe the data.

### Scratch assay

HCT116 or HT29 cells were grown in six-well plates until 70% confluence, and wounds were created with 200 μL pipette tips. Samples were evaluated at 0, 24, 48, and 72 h after scratching. The wound healing conditions were assessed and recorded.

### RNA immunoprecipitation

RNA immunoprecipitation was performed using EZMagna RIP Kit (Millipore, USA). The HCT116 cell lysates were incubated with 5 μg Argonaute-2 (Ago2) antibody (Abcam, USA) or a control anti-immunoglobulin G (IgG) antibody (Abcam, USA) coated beads at 4 °C overnight under rotation. Subsequently, total RNA was collected for analyzing the expression levels of mRNA and miRNA via RT-qPCR.

### RNA pulldown

Biotin was linked to the 3-end of NALT1 and miRNA-574-5p. HCT116 cells were resuspended in lysis buffer containing 100 mM KCl, 5 mM MgCl2, 20 mM Tris (pH7.5), 0.3% NP-40, protease inhibitor cocktail (Roche, USA), and 50U RNase OUT (Invitrogen, USA). The HCT116 lysates were incubated with Biotin-labeled NALT1 and miRNA-574-5p probes. The levels of lncRNA, miRNA, and mRNA in the pulldown of Biotin-NALT1 and miRNA-574-5p were quantified by real-time PCR.

### RNA fluorescence in situ hybridization

Cy3-labeled specific probe to NALT1 was designed and synthesized by RiboBio (lnc1CM001, RiboBio, China) and the intensities were determined by FISH Kit (C10910, RiboBio, China) by following the manufacturer’s protocol.

### In vivo model

BALB/c nude mice (male, 4-week-old) were supplied by the Animal Centre of Guangxi Medical University. All mice were raised under pathogen-free conditions. The in vivo study was conducted in compliance with the national and institutional guidelines and was approved by the Institutional Animal Care and Use Committee.

In the tumor growth xenograft model, the flanks of each mouse were subcutaneously injected with 1 × 10^6^ viable HCT116 cells (*n* = 4 for each group). After 2 weeks, the tumors were collected and paraffin-embedded, then subjected to H&E staining or immunohistochemistry (IHC). Tumor volume was calculated as (*W*^2^/2 × *L*, *W* and *L* denote the width and length of the tumor, respectively).

In the metastasis model, 2 × 10^6^ viable HCT116 cells were injected into the spleen of BALB/c nude mice (*n* = 4 for each group). After 5 weeks of injection, the lungs were collected for further study. Metastatic nodules of the lung were counted and paraffin-embedded and subjected to H&E staining.

### Statistical analysis

All statistic tests were conducted using the GraphPad Prism 5.0 or the SPSS 25 software. For continuous variables, the data are presented as mean ± SEM. Student’s *t* test (unpaired, two-tailed) or one-way ANOVA was employed to determine the means between two or multiple groups. The correlations among lncRNA NALT1, miRNA-574-5p, and PEG10 were determined by Spearman’s rank test. *P* < 0.05 was deemed statistically significant.

## Results

### NALT1 is a noncoding RNA localized in nucleus and cytoplasm, which can be an oncogene in CRC

**Fig. 1:**
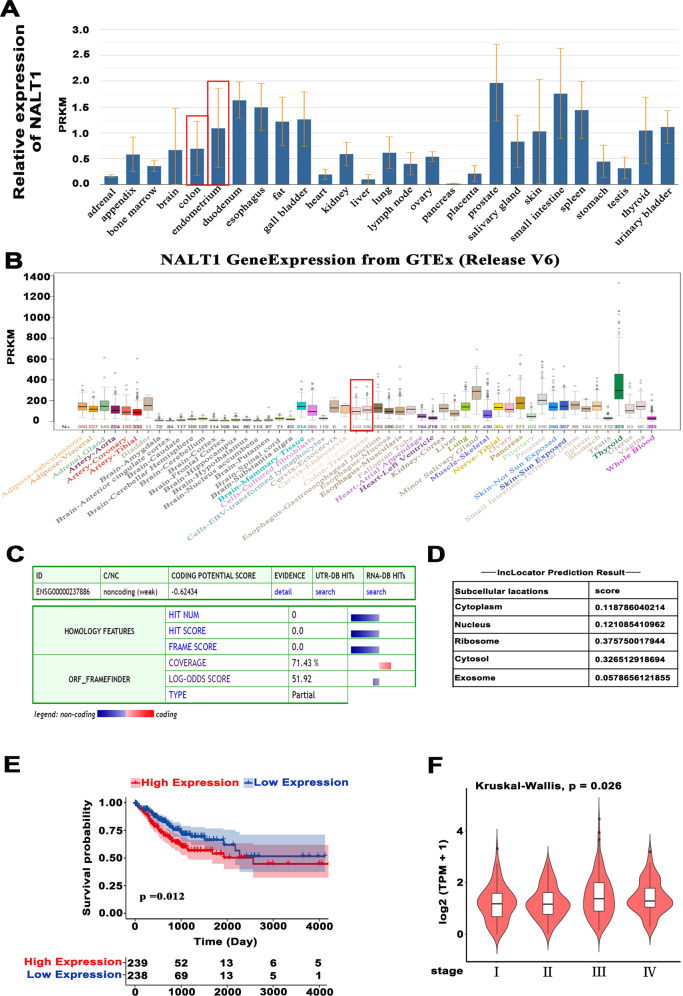
NALT1 is a noncoding RNA localized in nucleus and cytoplasm that might be an oncogene in CRC. **A**, **B** Expression of NALT1 in normal tissues derived from UCSC and NCBI. **C** CPC assessed that NALT1 has lost its protein-coding capability. **D** lncLocator was applied to estimate the subcellular localization of NALT1. **E** Patients with high NALT1 expression survived shorter than those with low NALT1 expression. **F** NALT1 expression was increased during the high-risk stage.

Given that NALT1 has been rarely studied, its specific functions, such as protein‐coding ability, expression patterns, localization, and survival correlation, remain unknown. Based on the data collected from NCBI (https://www.ncbi.nlm.nih.gov/gene/?term=NALT1), NALT1 was typically overexpressed in the majority of normal tissues, including normal intestinal tissues, except for normal adrenal, heart, liver, pancreas, and placenta (Fig. 1A). Moreover, the data from UCSC (http://genome.ucsc.edu) also showed a high expression of NALT1 in 53 human normal tissues and highest median expression in colon-sigmoid (Fig. 1B). Additionally, the online Coding Potential Assessment Tool (CPC; http://cpc.gao-lab.org/programs/run_cpc.jsp) assessed that NALT1 was not able to encode a protein (Fig. 1C). Besides, the online lncLocator (http://www.csbio.sjtu.edu.cn/bioinf/lncLocator/) was also used to determine the subcellular localization of NALT1 in cells. The data revealed that NALT1 was distributed in both the nucleus and cytoplasm (Fig. 1D). In addition, the GEPIA database (http://gepia2.cancer-pku.cn/#analysis) indicated that patients with high NALT1 expression survived shorter than those with low NALT1 expression (Fig. 1E) and expression of NALT1 increased along with the stages of CRC progression (Fig. 1F).

### Down-regulation of NALT1 suppressed CRC proliferation, migration, and invasion in vitro

**Fig. 2:**
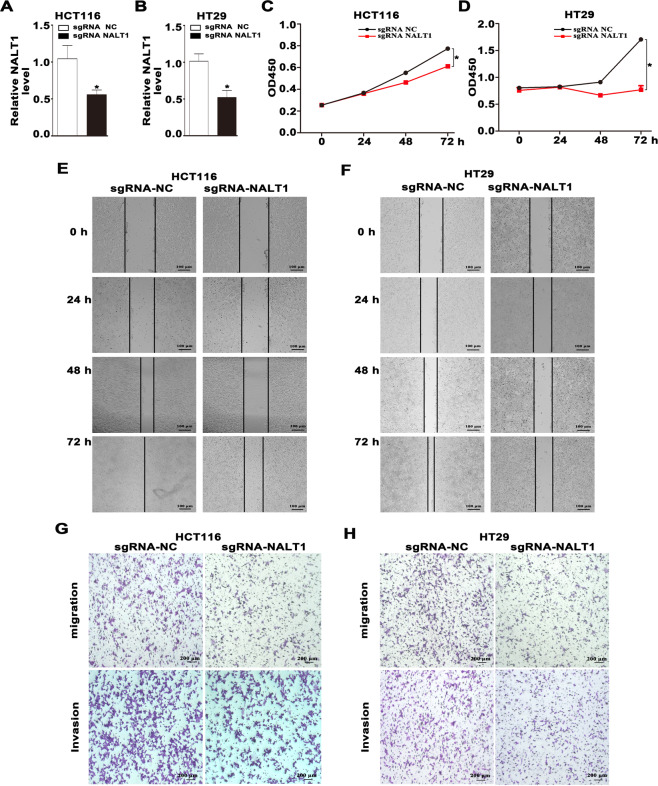
Down-regulation of NALT1 suppressed CRC proliferation, migration, and invasion in vitro. **A**, **B** The expression of NALT1 in HCT116 and HT29 cells with NALT1 knockdown. GAPDH was utilized as the loading control. **C**, **D** The viability curves of HCT116 and HT29 cells with NALT1 knockdown were determined by CCK-8 assay at different time points. **E**–**H** Representative images of wound healing, migration, and invasion assays of HCT116 and HT29 cells with NALT1 knockdown.

To verify the specific roles of NALT1 in CRC, the functions of NALT1 in CRC cells were investigated. It was found that NALT1 was remarkably upregulated in CRC cell lines HCT116 and HT29 compared with the normal cell line NCM460 (Fig. S1A). We used HCT116 and HT29 cell lines to conduct the loss-of-function tests. As shown in Fig. 2A, B, by using CRISPR/Cas9 technology, the expression of NALT1 was dramatically reduced in both HCT116 and HT29 cells. Additionally, silencing NALT1 could reduce the viability of HCT116 and HT29 cells (Fig. 2C, D). Further, wound healing assays revealed that after NALT1 silencing, the gap closure rate reduced in both HCT116 and HT29 cells (Fig. 2E, F). Moreover, transwell assays showed that NALT1 knockdown significantly inhibited the migratory and invasive capacity of HCT116 and HT29 cells (Fig. 2G, H).

### Up-regulation of NALT1 promotes CRC proliferation, migration, and invasion in vitro

**Fig. 3:**
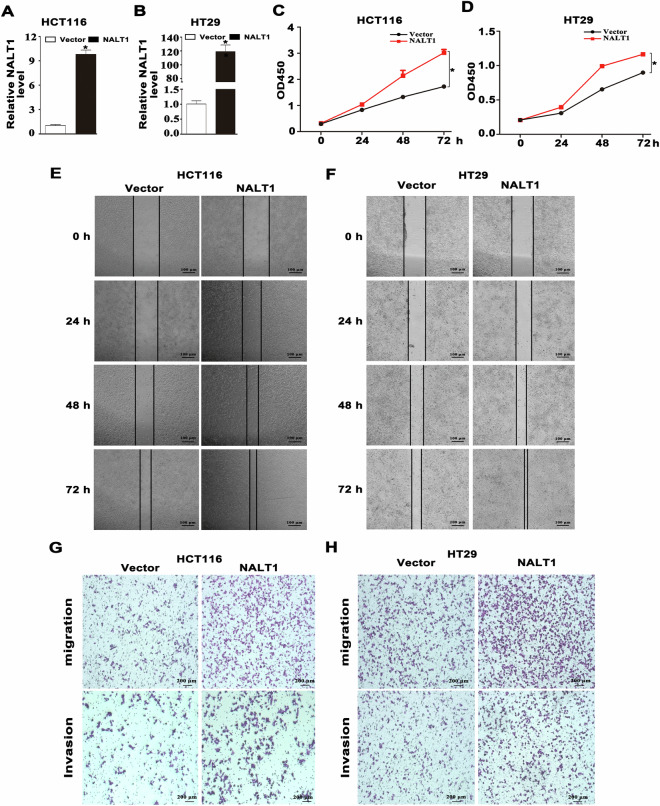
Up-regulation of NALT1 promotes CRC proliferation, migration, and invasion in vitro. **A**, **B** The expression of NALT1 in HCT116 and HT29 cells with NALT1 overexpression. GAPDH was utilized as the loading control. **C**, **D** The viability curves of HCT116 and HT29 cells with NALT1 overexpression were determined by CCK-8 assay at different time points. **E**–**H** Representative images of wound healing, migration, and invasion assays and of HCT116 and HT29 cells with NALT1 overexpression.

After transfection with a NALT1-overexpressed plasmid-containing vector, the expression of NALT1 in HCT116 and HT29 cells was greatly elevated compared to the empty vector group (Fig. 3A, B). The cell proliferation was decreased compared to the empty vector group based on the results of CCK8 assays (Fig. 3C, D). Additionally, wound healing assays revealed that after NALT1 overexpression, the gap closure rate increased in both HCT116 and HT29 cells (Fig. 3E, F). Moreover, the migratory and invasive capacity of HCT116 and HT29 cells transfected with NALT1 overexpression plasmids were significantly enhanced (Fig. 3G, H).

### NALT1 promotes PEG10 expression in CRC

**Fig. 4:**
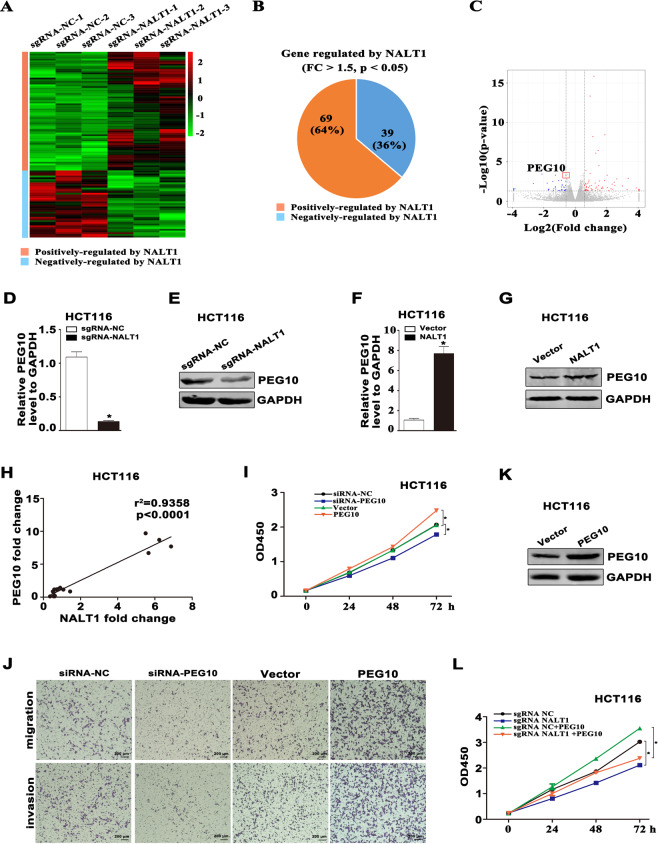
NALT1 promotes PEG10 expression in CRC. **A** Heatmap revealing the significantly differentially expressed mRNA in NALT1-knockdown and NC HCT116 cells. Green and red denote downregulated and upregulated mRNAs, respectively. **B** Genes were negatively and positively regulated by NALT1, respectively (fold-change>1.5, *q* < 0.05). **C** Ten top genes regulated by NALT1 with fold-change>1.5 and p<0.05 are drawn in the volcano plot. **D**–**G** RT-qPCR and WB analyses of PEG10 expression after knockdown or overexpression of NALT1 in HCT116 cells. **H** Gene expression correlation between NALT1 (x) and PEG10 (y) in HCT116 cells (*R* = 0.2106, *p* = 0.0317). **I** CCK8 assay was applied to measure the cell proliferation in PEG10-knockdown or overexpression HCT116 cells. **K** WB analyses the efficiency of PEG10 eukaryotic expression plasmid in HCT116 cells. **J** Transwell assays were applied to measure the migratory and invasive capability of HCT116 cells with PEG10 knockdown or overexpression. **L** CCK8 assay was applied to measure the cell proliferation in NALT1-knockdown or NC HCT116 cells after transfection with PEG10 overexpression plasmid or control.

To identify the key genes and signaling pathways mediated by NALT1, RNA sequencing (RNA-seq) was conducted, and the expression profiles of NALT1-KO and NALT1-control were compared. The effect of NALT1 on gene expression was consistent among the three biological repeats (Fig. S1B). The expression patterns of these NALT1-mediated genes were revealed by heatmap (Fig. 4A). Notably, there were 39 and 69 genes negatively and positively regulated by NALT1, respectively (fold-change > 1.5, *p*-value < 0.05; Fig. 4B). To verify our RNA-seq results and assess their roles in CRC, the top ten candidate genes (log2 fold-change > 1.5) were analyzed in CRC cells (Fig. 4C). Among these genes, gene paternally expressed 10 (PEG10) holds a high transcript abundance with the lowest *p*-value (*p* = 0.000544). Based on these characteristics, PEG10 was chosen for our subsequent analysis. It was observed that both mRNA and protein expression of PEG10 was downregulated after silencing NALT1 and upregulated after overexpressing NALT1 (Figs. 4D–G and S1C, D). The NALT1 level was highly correlated with the level of PEG10 in HCT116 and HT29 cell lines (Figs. 4H and S1E). Interestingly, cell proliferation, migration, and invasion assays showed that PEG10 knockdown significantly inhibited the proliferative, migratory, and invasive capacity of HCT16 and HT29 cells, whereas PEG10 overexpression exhibited an opposite trend in both HCT116 and HT29 cells (Figs. 4I, J and S1F). However, it remains unknown whether PEG10 is responsible for the process of NALT1‐mediated CRC development. Then, we constructed a PEG10 eukaryotic expression plasmid and verified overexpression efficiency (Figs. 4K and S1G). Interestingly, ectopic expression of PEG10 increased NALT1 knockdown-mediated inhibition of viability in HCT116 and HT29 cells (Figs. 4L and S1H).

### NALT1 serves as a sponge of miRNA-574-5p

**Fig. 5:**
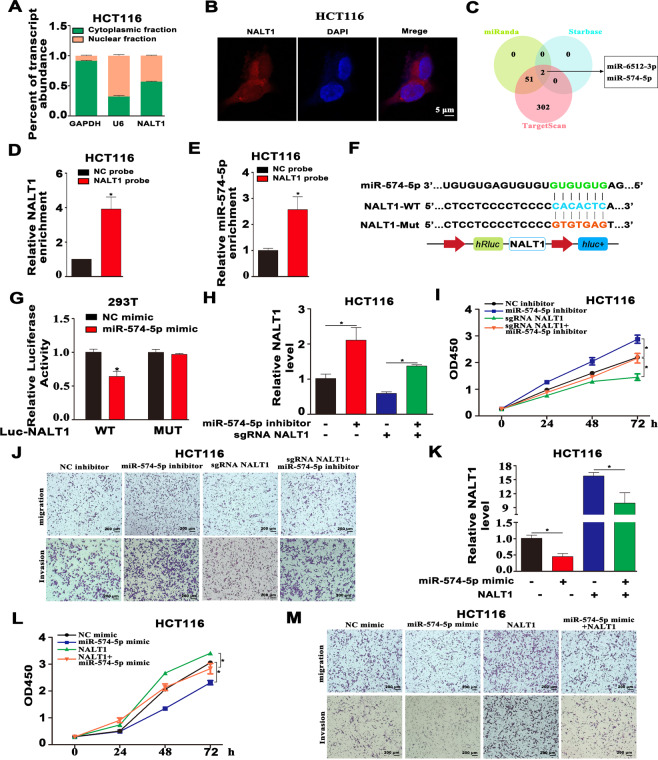
NALT1 serves as a sponge of miRNA-574-5p. **A**, **B** Subcellular localization analysis of NALT1 in HCT116 cells. Blue, DAPI; Red, NALT1. **C** Two miRNAs targeted by NALT1 were estimated by cross-analysis with TargetScan, Starbase, and miRanda databases. **D** The efficiency of the biotinylated NALT1 probe in HCT116 cells was verified by RT-qPCR. **E** The relative expression of miRNA-574-5p enriched by the NALT1 probe in HCT116 cells was evaluated by RT-qPCR. **F** Schematic diagram of Mut-NALT1 and WT-NALT1 LR vectors. **G** HCT116 was cotransfected with miRNA-574-5p mimic and Mut or WT NALT1 LR vectors, and the activities of the LR vectors were detected. **H**, **I** RT-qPCR and CCK8 assays were conducted to measure the cell proliferation in NALT1-knockdown or NC HCT116 cells after transfection with miRNA-574-5p inhibitor or NC. **J** Transwell assays were conducted to measure the migration and invasion capability in NALT1-knockdown or NC HCT116 cells after transfection with miRNA-574-5p inhibitor or NC. **K**, **L** RT-qPCR and CCK8 assays were performed to measure the NALT1 level and cell proliferation in NALT1-overexpression or NC HCT116 cells after transfection with miRNA-574-5p mimic or NC. **M** Transwell assays were conducted to measure the migration and invasion capability in NALT1-overexpression or NC HCT116 cells after transfection with miRNA-574-5p mimics or NC.

We next explored the specific mechanism by which NALT1 regulates PEG10 to affect CRC progression. Accumulating evidences have confirmed that cytoplasmic lncRNA can act as a miRNA sponge to modulate the expression of downstream target gene. As manifested in Figs. 5A and S1I, NALT1 was both located in the nucleus and cytoplasm of HT29 and HCT116 cells, while mainly in the cytoplasm. In agreement with the data of cellular fractionation assay, NALT1 was mainly localized in cytoplasm as indicated by RNA-fluorescence in situ hybridization (FISH; Figs. 5B and S1J). The target recognition sequences of NALT1, PEG10, and miRNA were predicted through bioinformatics analysis (TargetScan, Starbase, and miRanda). In total, two miRNAs (miRNA-574-5p and miRNA-6512-3p) emerged as potential targets for NALT1 and PEG10 in all of the databases (Fig. 5C). Studies have reported miRNA-574-5p both as oncogene or tumor suppressor gene in CRC [19, 20], so we sought to clarify the function of miRNA-574-5p in CRC. Firstly, RNA pull-down assay was performed to confirm the binding partner of NALT1. The efficiency of the biotin-labeled NALT1 probe was verified by RT-qPCR (Fig. 5D). The results showed that miRNA-574-5p was highly enriched by the NALT1 probe in the HCT116 cell line (Fig. 5E). Next, the putative binding sites of NALT1 and miRNA-574-5p were presented (Fig. 5F). To determine the interactions between NALT1 and miRNA-574-5p, a luciferase reporter (LR) containing mutant (Mut) or wild-type (WT) NALT1 cDNA sequences was constructed according to the putative binding sites of NALT1 and miRNA-574-5p. The LR assays revealed that miRNA-574-5p mimic transfection inhibited the luciferase activities in cells containing NALT1 WT reporter, whereas no obvious difference was found in cells containing NALT1 Mut reporter (Fig. 5G). In the rescue experiment, the downregulation of NALT1 in HCT116 and HT29 cells reversed the increased expression of NALT1 and enhancement of cell proliferation, migration, and invasion induced by miRNA-574-5p inhibitor (Figs. 5H–J and S1K-L). Meanwhile, NALT1 overexpression reversed the downregulation of NALT1 and inhibition of cell proliferation, migration, and invasion induced by miRNA-574-5p mimic (Figs. 5K–M and S1M, N).

### PEG10 is a direct target of miRNA-574-5p

**Fig. 6:**
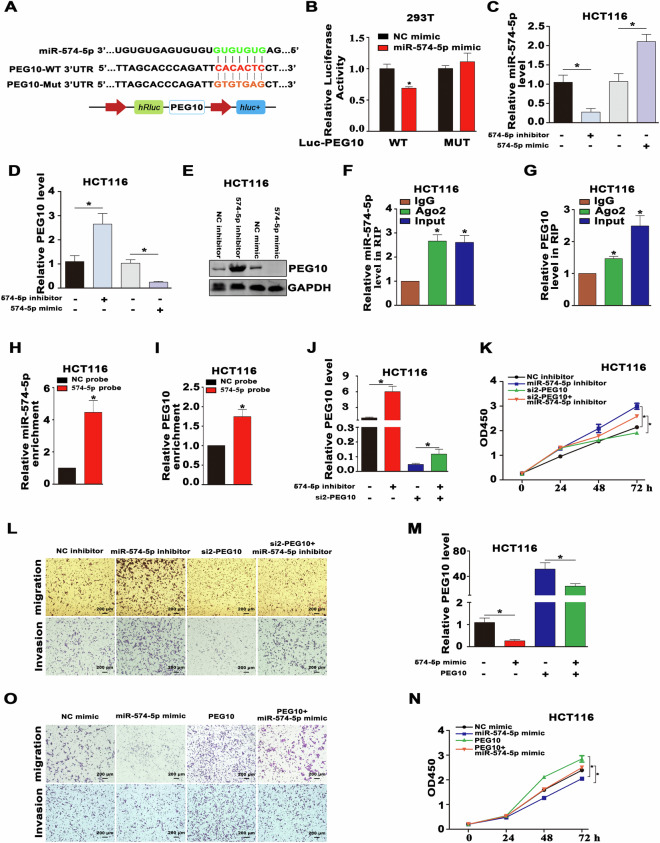
PEG10 is a direct target of miRNA-574-5p. **A** Schematic diagram of Mut-PEG10 3′UTR and WT-PEG10 3’UTR LR vectors. **B** 293T was cotransfected with miRNA-574-5p mimics and Mut or WT PEG10 3′UTR LR vectors, and the activities of the LR were detected. **C**, **D** The mRNA levels of miRNA-574-5p and PEG10 were assessed by RT-qPCR in CRC cells transfected with miRNA-574-5p inhibitor or mimic. **E** The protein level of PEG10 was assessed by WB in CRC cells transfected with miRNA-574-5p inhibitor or mimic. **F**, **G** Ago2 RIP was conducted to detect endogenous Ago2 binding to RNA; IgG was employed as the control. The levels of miRNA-574-5p and PEG10 were detected by RT-qPCR, and shown as fold-enrichment in Ago2 compared to lgG. **H** The efficiency of the biotinylated miRNA-574-5p probe in HCT116 cells was verified by RT-qPCR. **I** The relative expression of PEG10 enriched by the miRNA-574-5p probe in HCT116 cells was determined by RT-qPCR. **J**–**L** RT-qPCR, CCK8, and transwell assays were conducted to measure the expression of PEG10 and cell proliferation, migration, and invasion in HCT116 cells after cotransfection with miRNA-574-5p inhibitor, control and PEG10 siRNA. **M**–**O** RT-qPCR, CCK8, and transwell assays were conducted to measure the expression of PEG10 and cell proliferation, migration, and invasion in HCT116 cell line after cotransfection with miRNA-135b mimic, control, and PEG10 overexpression plasmid.

The RNA-induced silencing complex (RISC) is generated from a miRNA ribonucleoprotein complex (miRNP) present in anti-Ago2 immunoprecipitates. We found that there were putative binding sites between PEG10 and miRNA-574-5p (Fig. 6A). The LR assays showed that the miRNA-574-5p mimic reduced the luciferase activities of 3′-UTR of WT PEG10 group, but not the 3′-UTR of MUT PEG10 group (Fig. 6B). Next, we used both Western blot (WB) and RT-qPCR assays to confirm that miRNA-575-5p mimic markedly reduced the mRNA and protein expression of PEG10 in HCT116 and HT29 cells, whereas miRNA-575-5p inhibitor exhibited an opposite trend (Figs. 6C–E and S2A, B). Ago2-RIP assay was conducted using anti-Ago2 in HCT116 extracts, and the results showed that PEG10 and miRNA-574-5p were enriched significantly in Ago2-containing miRNPs compared to anti-IgG immunoprecipitates (Figs. 6F, G and S2C, D). In addition, RNA pull-down assay was conducted to confirm the binding partner of miRNA-574-5p, and the efficiency of the biotin-labeled miRNA-574-5p probe was verified by RT-qPCR (Fig. 6H). The results showed that PEG10 were highly enriched by the miRNA-574-5p probe in HCT116 cell lines (Fig. 6I). We designed siRNAs targeting different domains of PEG10 and the siRNAs knockdown efficiency was verified by qRT-PCR and WB in HCT116 and HT29 cells (Fig. S2E–H). Furthermore, knockdown of PEG10 by siRNA reversed miRNA-574-5p inhibitor-mediated enhancement of PEG10 expression, cell proliferation, migratory, and invasive ability (Figs. 6J–L, and S2I–J. Also, overexpression of PEG10 reversed miRNA-574-5p mimic-mediated inhibition of PEG10 expression, cell proliferation, migratory, and invasive ability (Fig. 6M–O).

### In vivo examination of the impact of NALT1 on cell growth and metastasis capability

**Fig. 7:**
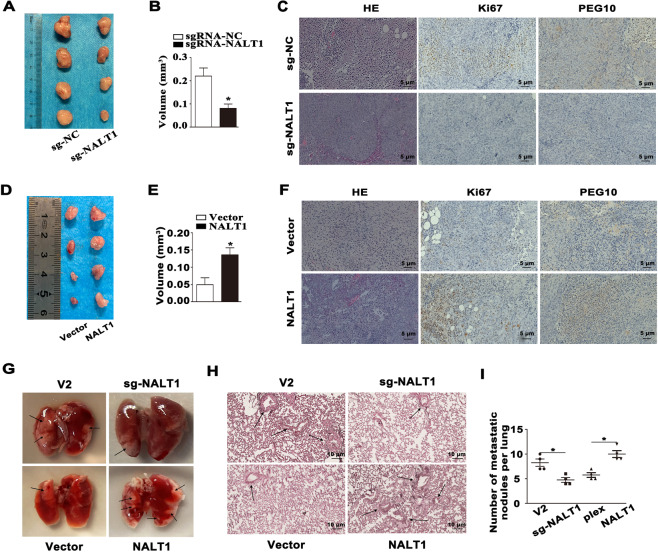
In vivo examination of the impact of NALT1 on cell growth and metastasis capability. **A** Representative images of the tumors after subcutaneous injection with NALT1-knockdown and NC HCT116 cells. **B** Tumor volume was analyzed in NALT1-knockdown/control group. **C** Representative images of immunofluorescence and H&E staining using antibodies against PEG10 and Ki-67. **D** Representative images of the tumors after subcutaneous injection with NALT1-overexpression and NC HCT116 cells. **E** Tumor volume was analyzed in the NALT1-overexpression/control group. **F** Representative images of immunofluorescence and H&E staining using antibodies against PEG10 and Ki-67. **G** After injection of NALT1-knockdown or overexpression HCT116 cells into the spleen of nude mice at five weeks, the lungs were isolated from the mice and the metastatic lung nodules were marked. **H** Representative images of lung tissue sections by H&E staining. **I** The number of metastatic nodules in the lungs.

In the tumor growth xenograft model, HCT116 cells were injected subcutaneously into nude mice (*n* = 4 for each group). After 2 weeks of injection, the mice were killed and tumors were obtained. We found that knockdown of NALT1 decreased tumor volume and inhibited tumor growth compared with the control group (Figs. 7A, B and S2K, L). Moreover, IHC and H&E staining on paraffin-embedded specimens of xenograft tumors indicated that the levels of PEG10 and Ki67 were remarkably downregulated in the xenograft tumors with NALT1 knockdown compared with the control group (Fig. 7C). Meanwhile, overexpression of NALT1 significantly promoted tumor growth compared with the control group (Figs. 7D, E and S2M, N). The levels of PEG10 and Ki67 were markedly upregulated in the NALT1-overexpressed tumors compared with the control group (Fig. 7F).

To investigate the metastatic potential of NALT1 in vivo, HCT116 cells were injected into the spleen of nude mice (*n* = 4 for each group). Five weeks after injection, the lungs were collected and H&E staining was performed to analyze the lung metastatic lesions. The results showed that knockdown of NALT1 decreased the number and size of lung metastatic lesions. Also, overexpression of NALT1 aggravated lung metastatic lesions (Fig. 7G–I).

### NALT1 may serve as a promising prognostic biomarker for CRC

**Fig. 8:**
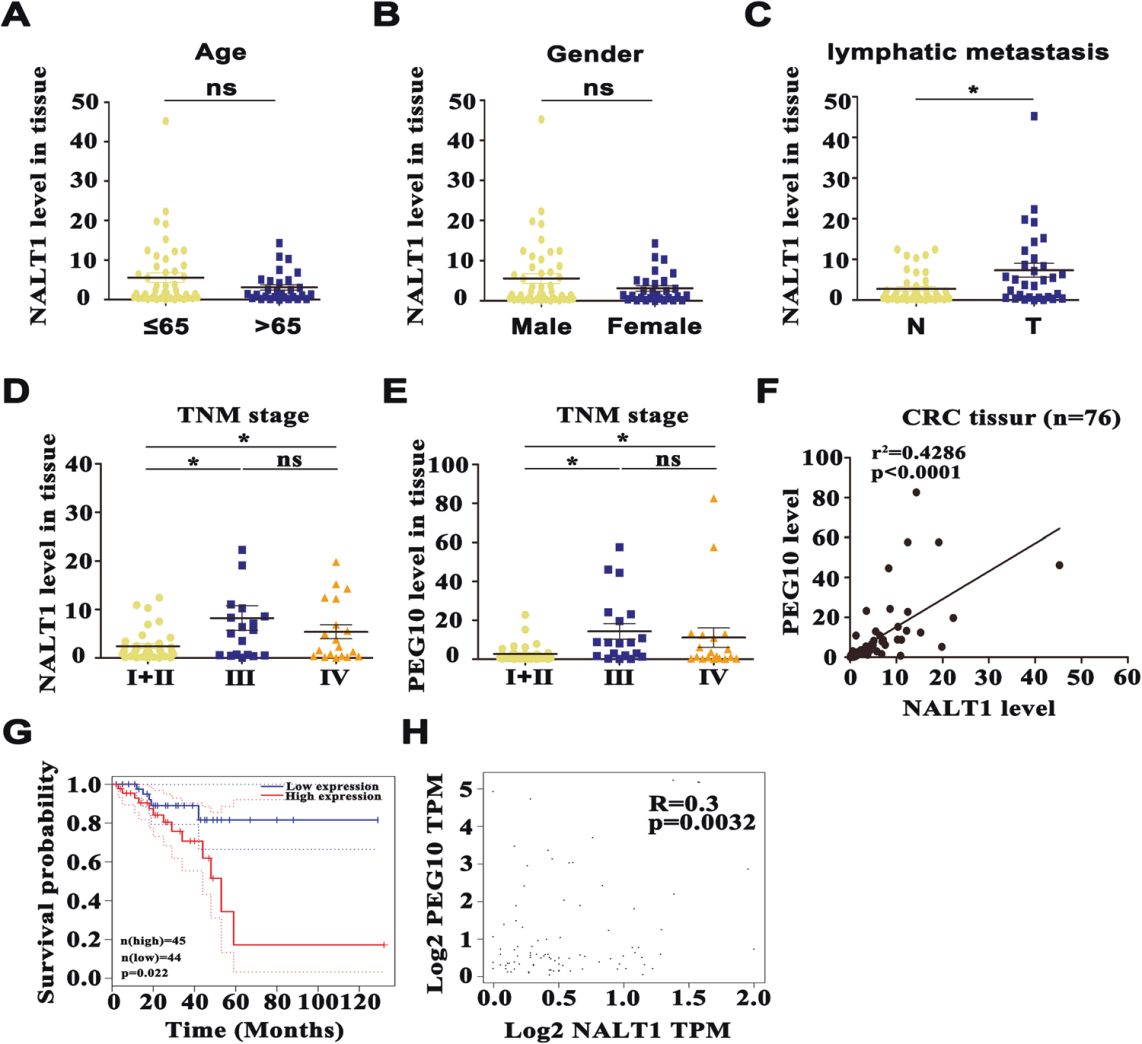
NALT1 may serve as a promising prognostic biomarker for CRC. **A**–**E** Predictive value of NALT1 tissue expression in clinicopathological features based on the CRC cohort (*n* = 76). **F** Tissue expression of NALT1 was associated with that of PEG10 in the CRC cohort (*n* = 76). Mean ± SD. Student’s *t* test or ANOVA. *p < 0.05; ns not significant. **G** Patients with rectum adenocarcinoma with high PEG10 expression survived shorter than those with low PEG10 expression in the GEPIA database. **H** The expression of NALT1 was associated with that of PEG10 in the CRC cohort in the GEPIA database.

**Fig. 9:**
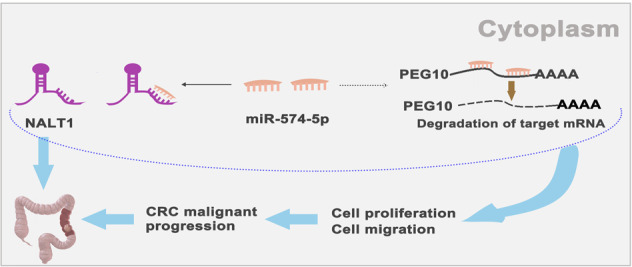
Schematic representations of pathways modulated by NALT1 in CRC. NALT1 is upregulated in advanced-stage CRC and promotes CRC progression via a ceRNA pattern by sponging miR-574-5p, which facilitates PEG10 expression.

To further investigate the clinicopathological features and prognostic values of NALT1 expression in CRC patients, 76 different TNM stages CRC tissues (ranging from stages I through IV, all of them had detailed clinical and follow-up information available) were evaluated. Multivariate regression revealed that high level of NALT1 was related to differentiation (*p* < 0.001), distant metastasis (*p* = 0.0085), and tumor size (*p* = 0.0069; Table 1). On the contrary, age and gender were not associated with tissue expression of NALT1 (Table 1 and Fig. 8A, B). Lymphatic metastasis was associated with tissue expression of NALT1 (Fig. 8C). Stage III/IV patients had higher tissue levels of NALT1 and PEG10 than stage I/II patients (Fig. 8D, E). Furthermore, the correlation between NALT1 and PEG10 expression was also examined. As displayed in Fig. 8F, an obvious positive correlation was observed between NALT1 and PEG10 in CRC patients. Accordingly, the GEPIA database (http://gepia2.cancer-pku.cn/#analysis) indicated that patients with rectum adenocarcinoma with high PEG10 expression survived shorter than those with low PEG10 expression (Fig. 8G). Also, an obvious positive correlation was observed between NALT1 and PEG10 in patients with rectum adenocarcinoma by the GEPIA database (Fig. 8H and Fig. 9).

### Schematic diagram

(Figure 9).

## Discussion

In recent years, lncRNAs have been found to be associated with CRC. Numerous lncRNAs in the human genome have improved our understanding of human diseases, including cancers. Dysregulated lncRNAs in the tumor have been used as molecular markers for predicting CRC progression. For example, lncRNA MIR17HG was specifically highly expressed in liver metastatic CRC tissues and predicted poor survival in patients with liver metastasis [11]. Furthermore, the vital function and mechanism of lncRNAs in tumorigenesis and progression have been recognized with the help of clinical bioinformatics analysis combined with functional research. Previous studies had identified the oncogenic role of NALT1 in GC tissues and related to poor patients’ prognosis [4, 5]. Very little information is available about the molecular mechanisms of NALT1 in CRC carcinogenesis. In the present study, we confirmed, for the first time (to our knowledge), the oncogenic function of NALT1 in CRC and the underlying mechanism by which NALT1 facilitates the malignant proliferation and metastasis of CRC cells. Importantly, NALT1 level was associated with the advanced CRC stage. Thus, NALT1 might be a promising target for diagnosis of CRC.

LncRNAs mediate their function through various mechanisms, including scaffolding molecules, co-transcriptional regulations, sponge molecules, etc. While their biological functions are often dependent on the specific intracellular localization. Among these, the ceRNA hypothesis introduced by scholars at Harvard University in 2011 has received considerable attention, which posited that there is an interactive mode among lncRNA, miRNA, and mRNA, and lncRNAs can modulate mRNA via a competitive miRNA inhibition with the presence of miRNA response elements (MRE). For instance, lncRNA ITGB8-AS1 can function as a ceRNA to induce CRC proliferation and migration via regulating focal adhesion signaling [10]. In this work, NALT1 was observed in both the cytoplasm and nucleus and mainly in the cytoplasm.

Mechanismly, NALT1 acts as a ceRNA for miRNA-574-5p, leading to enhanced expression of PEG10, which in turn facilitates proliferation, migration, and invasion of CRC cells in vitro and in vivo. A previous study has shown that miRNA-574-5p could serve as a tumor suppressor in CRC [19]. Another study uncovered that miRNA-574-5p suppressed breast cancer cells’ growth, migration, and EMT [21]. In this study, miRNA-574-5p downregulation partially restored the effects of NALT1 on CRC cell proliferation, tumor growth, and migration. Previous reports showed that miRNA-574-5p was involved in the ceRNA regulation network of cancer [22, 23], which was consistent with our findings. Thus, our findings may prove novel insights into the pathogenesis and treatment of CRC.

Meanwhile, we also proved that PEG10 was a downstream target of miRNA-574-5p. PEG10 was a famous oncogene and a downstream target of miRNA-27a-3p and miRNA-34a-5p in hepatocellular carcinoma [18], as well as, PEG10 regulated endometrial cancer and bladder cancer [16, 17]. Besides, PEG10 was regarded as a prognostic biomarker in patients with solid tumors [24]. Nonetheless, the functional role and underlying mechanism of PEG10 in CRC remain unclear. Interestingly, our results showed that PEG10 expression was closely related to the CRC tumor stage and survival, as well as positively correlated with NALT1 levels in CRC tissue. PEG10 could positively regulate the growth, migration, and invasion of CRC cells. In addition, PEG10 expression was reduced by enhanced miRNA-574-5p and was elevated by upregulated NALT1. Furthermore, PEG10 knockdown or overexpression partially rescued the effects of miRNA-574-5p and NALT1 on CRC cell proliferation, tumor growth, and migration, suggesting that PEG10 is essential for the NALT1/miRNA-574-5p-mediated biological effects in CRC cells. However, whether a regulation network exists related to NALT1, miRNA-574-5p, and PEG10 in early-stage CRC patients deserves further exploration.

In summary, the current study illustrated that NALT1 sponged miRNA-574-5p to facilitate late-stage CRC development, which was mediated by the upregulation of PEG10 in CRC cells. These findings offered a novel insight into the mechanisms underlying the role of NALT1 in late-stage CRC. Hence, NALT1/miRNA-574-5p/PEG10 axis might be a promising strategy for the diagnosis and treatment of colorectal tumors.

### Supplementary information

S1

S2

Original Data File

Reproducibility checklist

## Data Availability

The data used to support the findings of this study are available from the corresponding author upon request.
