# Correction: LncRNA NALT1 promotes colorectal cancer progression via targeting PEG10 by sponging microRNA-574-5p

**DOI:** 10.1038/s41419-024-06881-6

**Published:** 2024-07-17

**Authors:** Mengling Ye, Lu Zhao, Lu Zhang, Siyi Wu, Zhao Li, Yi Qin, Fei Lin, Linghui Pan

**Affiliations:** 1 Department of Experimental Research, Guangxi Medical University Cancer Hospital, Nanning, China; 2 Department of Anesthesiology, Guangxi Medical University Cancer Hospital, Nanning, China; 3 Guangxi Clinical Research Center for Anesthesiology, Nanning, China; 4 Guangxi Engineering Research Center for Tissue & Organ Injury and Repair Medicine, Nanning, China; 5 Guangxi Key Laboratory for Basic Science and Prevention of Perioperative Organ Disfunction, Nanning, China

**Keywords:** Colon cancer, Colon cancer

Correction to: *Cell Death and Disease* 10.1038/s41419-022-05404-5, published online 16 November 2022

In this article fig 3G and 6L have been given erroneously.

These errors were created during the preparation of the figures in this paper by copy and paste. The bottom row of Figure 3G (Invasion) was duplicated from Figure 2G. An image in Figure 6L (miR-574-5p inhibitor, invasion) was duplicated from an image in Figure 5M (NALT1, migration).
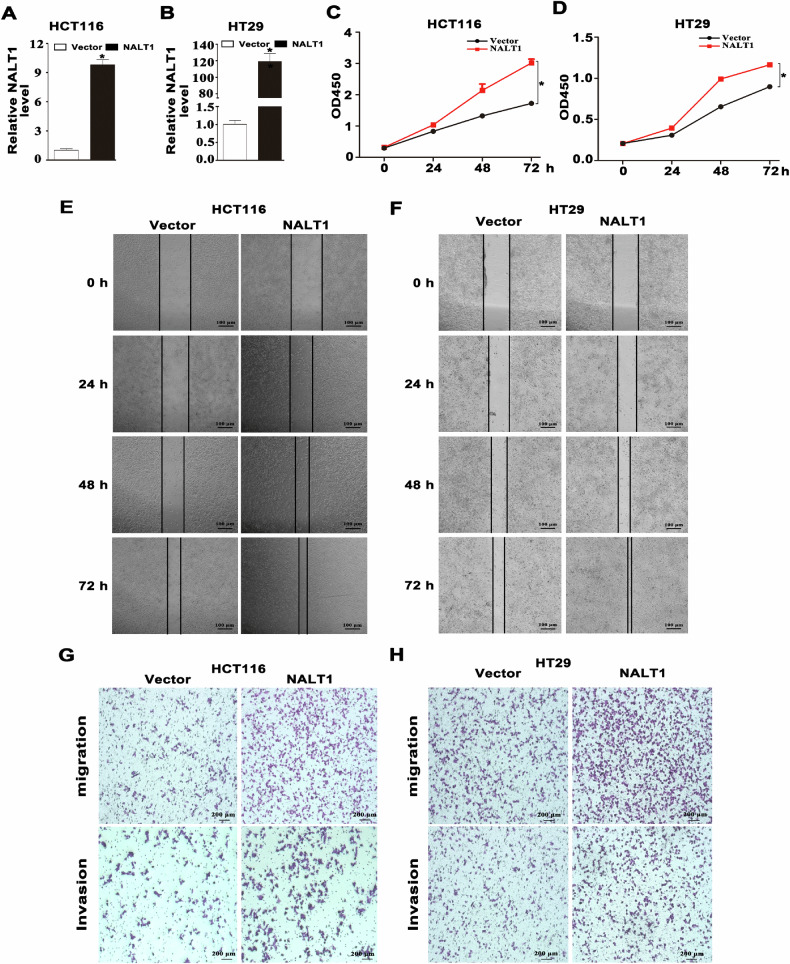

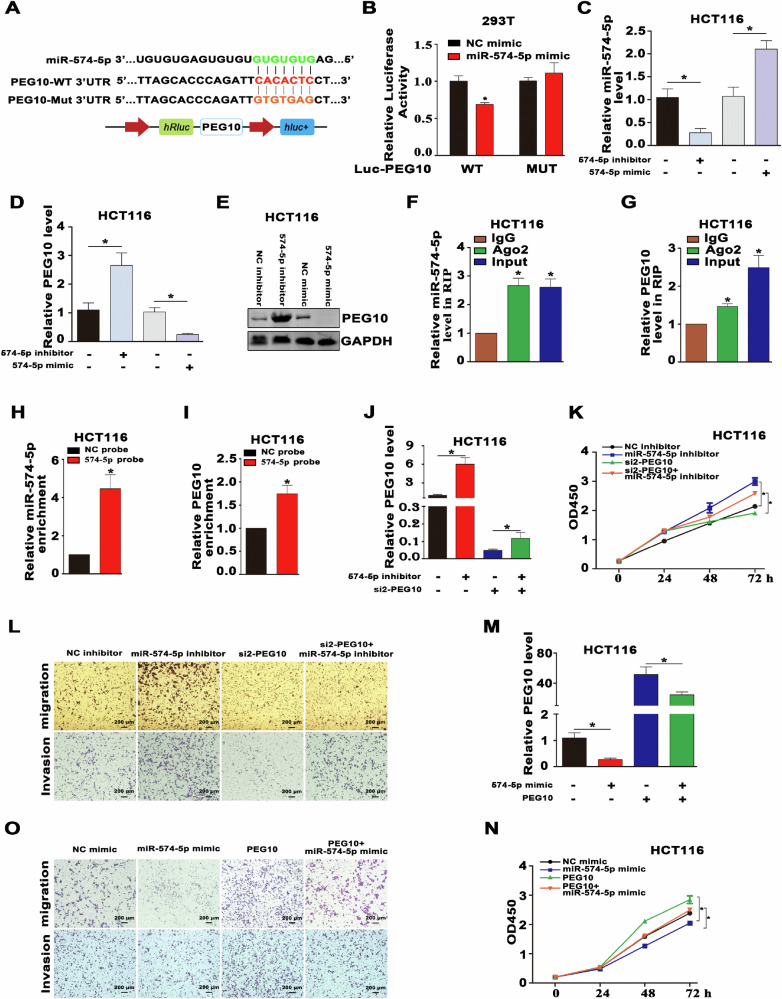


The original article has been corrected.

