# Supplementary figures and images for: LncRNA NALT1 promotes colorectal cancer progression via targeting PEG10 by sponging microRNA-574-5p
Source: Cell Death Dis. 2022 Nov 16;13(11):960. doi: 10.1038/s41419-022-05404-5 (PMC9669023; doi:10.1038/s41419-022-05404-5)

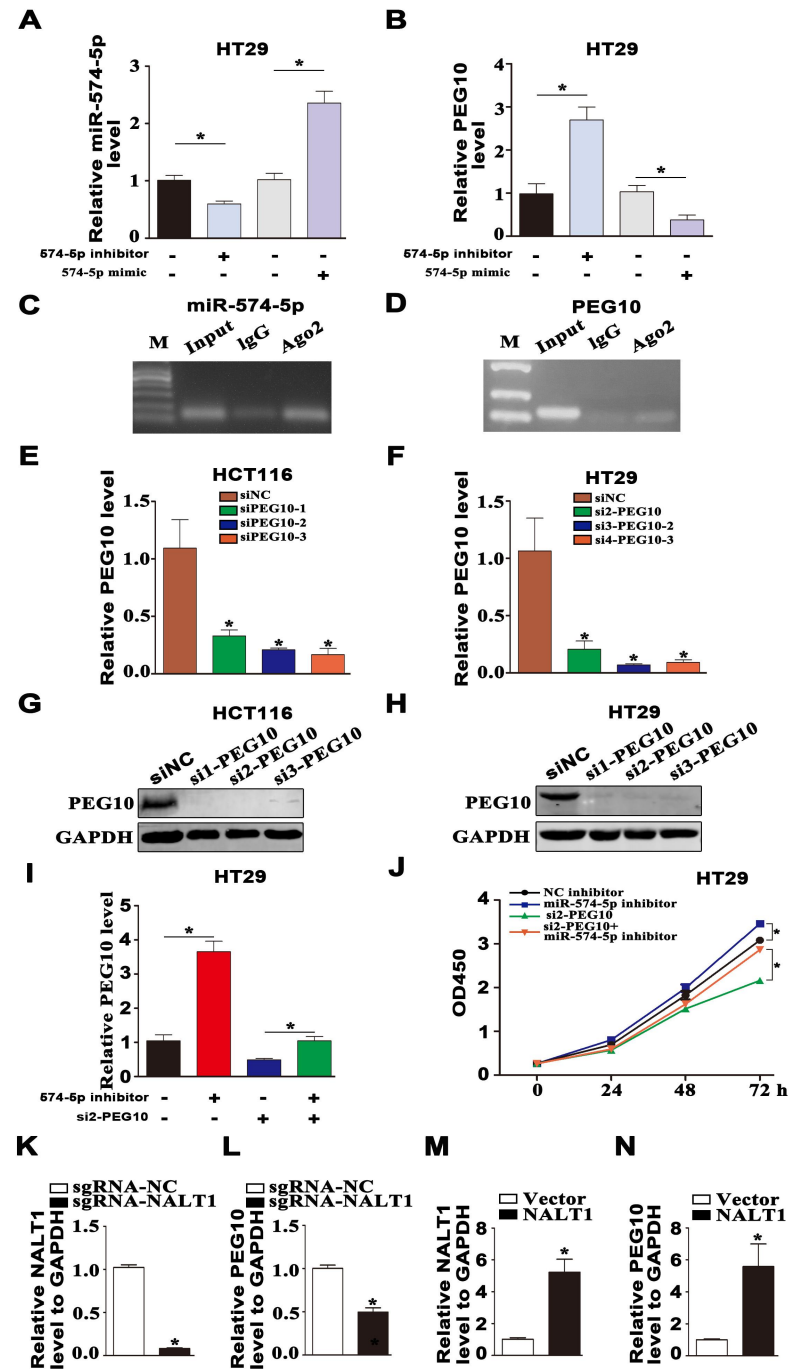

Supplement: Supplementary file 2 — S2 [file 41419_2022_5404_MOESM2_ESM.pdf]

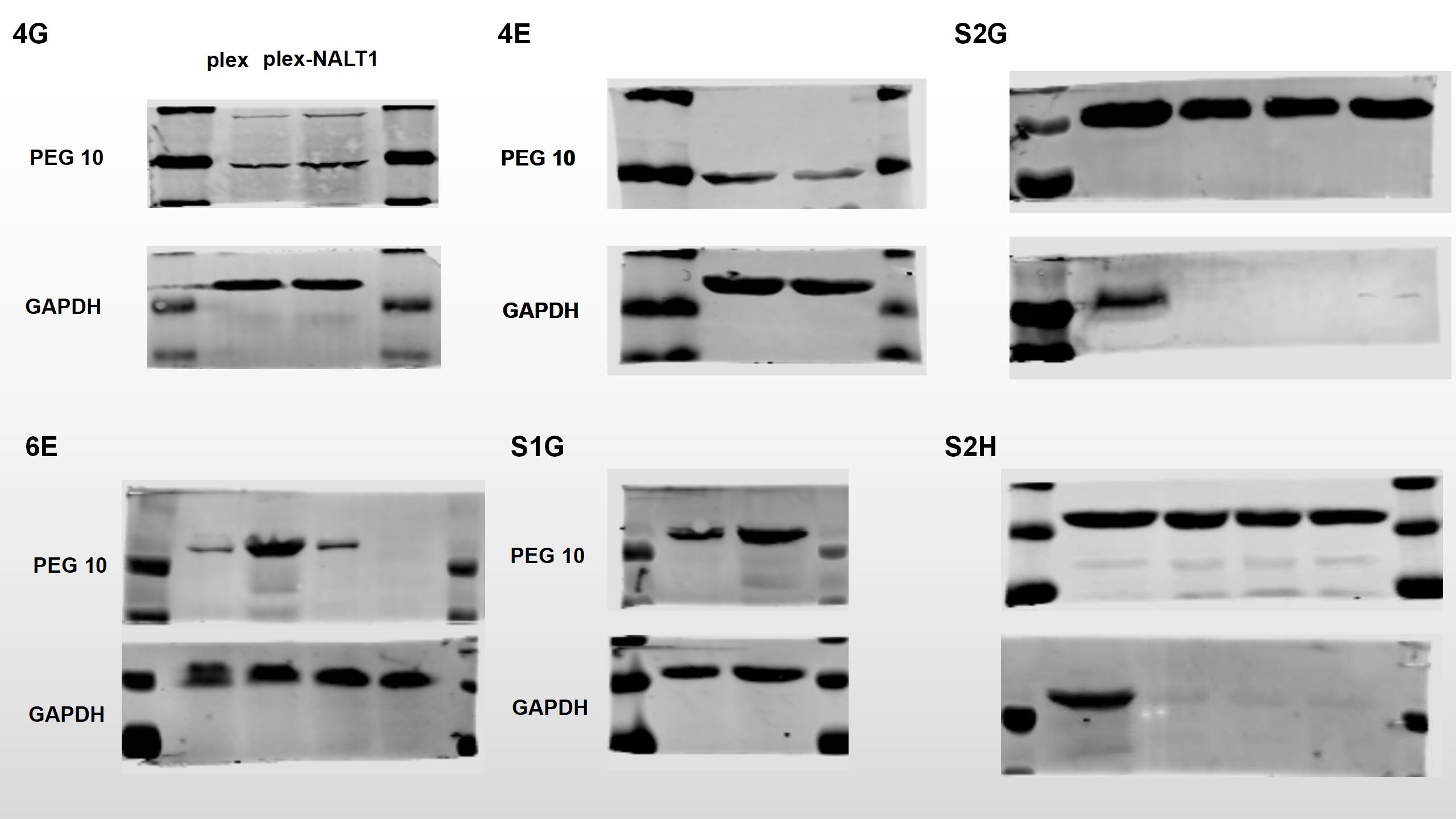

Supplement: Supplementary file 3 — Original Data File [file 41419_2022_5404_MOESM3_ESM.jpg]
